# DNA Barcoding Simplifies Environmental Risk Assessment of Genetically Modified Crops in Biodiverse Regions

**DOI:** 10.1371/journal.pone.0035929

**Published:** 2012-05-02

**Authors:** Chinyere V. Nzeduru, Sandra Ronca, Mike J. Wilkinson

**Affiliations:** 1 Biosafety Unit, Department of Forestry, Federal Ministry of Environment, Abuja, Nigeria; 2 Insitute of Biological Environmental and Rural Sciences, Aberystwyth University, Aberystwyth, Ceredigion, United Kingdom; University of Kentucky, United States of America

## Abstract

Transgenes encoding for insecticidal crystal (Cry) proteins from the soil-dwelling bacterium *Bacillus Thuringiensis* have been widely introduced into Genetically Modified (GM) crops to confer protection against insect pests. Concern that these transgenes may also harm beneficial or otherwise valued insects (so-called Non Target Organisms, NTOs) represents a major element of the Environmental Risk Assessments (ERAs) used by all countries prior to commercial release. Compiling a comprehensive list of potentially susceptible NTOs is therefore a necessary part of an ERA for any Cry toxin-containing GM crop. In partly-characterised and biodiverse countries, NTO identification is slowed by the need for taxonomic expertise and time to enable morphological identifications. This limitation represents a potentially serious barrier to timely adoption of GM technology in some developing countries. We consider Bt Cry1A cowpea (*Vigna unguiculata*) in Nigeria as an exemplar to demonstrate how COI barcoding can provide a simple and cost-effective means of addressing this problem. Over a period of eight weeks, we collected 163 insects from cowpea flowers across the agroecological and geographic range of the crop in Nigeria. These individuals included 32 Operational Taxonomic Units (OTUs) spanning four Orders and that could mostly be assigned to genus or species level. They included 12 Lepidopterans and two Coleopterans (both potentially sensitive to different groups of Cry proteins). Thus, barcode-assisted diagnoses were highly harmonised across groups (typically to genus or species level) and so were insensitive to expertise or knowledge gaps. Decisively, the entire study was completed within four months at a cost of less than 10,000 US$. The broader implications of the findings for food security and the capacity for safe adoption of GM technology are briefly explored.

## Introduction

Agriculture is one of the key driving forces of the Nigerian economy [1] and ranks second only to the oil industry for the generation of foreign exchange income [2]. This importance has led to an increasing desire to stimulate new growth into the Nigerian agricultural sector through technological advancement [3]. Nigeria has yet to sanction the commercial release of any GM crop, although the potential economic and environmental benefits afforded by the technology in neighbouring Burkina Faso and in South Africa [4] has stimulated re-examination of policy in this area [5].

Cowpea (*Vigna unguiculata (L.) Walp*) is widely cultivated in Nigeria [6], with its protein-rich seeds being used both for human consumption and animal feed [7]. The crop is highly resilient and is particularly well-adapted to drought stress; a feature that has made it especially popular in the drought-prone savannah regions of the tropics and subtropics [8]. However, cowpea also suffers from many pest problems [9], [10]. In Nigeria, the most notable of these is the Lepidopteran pod-borer, *Maruca vitrata*, which causes up to 80% yield losses annually [11]. Chemical insecticides have proved largely ineffective against this pest in the context of the small-scale farming that is most frequently practiced in Nigeria [12]. The possibility of adopting a biotechnological solution to the problem emerged recently following the production of a GM cowpea line that expresses the *Cry1AB* protein derived from *Bacillus Thuringiensis (Bt)* and is resistant to attack by *Maruca* [13].

As elsewhere in the world [14], a comprehensive Environmental Risk Assessment (ERA) is required prior to commercial release of these GM *Bt Cry1AB* cowpea plants in Nigeria. The possibility that potentially beneficial species (e.g. pollinators, predators or parasitoids of pests), or otherwise valued organisms (e.g. endangered or protected species) could be harmed by the presence of the Cry1 protein in the crop represents a key consideration of any ERA for GM events of this kind [15]. These unintended recipients of the transgene product are known collectively as Non Target Organisms (NTOs) and it is important that regulators have an understanding of the diversity of NTO species that could potentially be harmed by the transgene.

The individual importance of any NTO to the decision-making process could vary according to the value placed on the species or to its function, and on the probable scale and veracity of consequences from exposure to the Cry toxin. The high level of taxonomic specificity of the Cry proteins [16] means that the vast majority of NTOs are unlikely to be affected by its presence. However, this adage does not apply to NTOs that belong to the same taxonomic group as the targeted pest since these are highly likely to share the same susceptibility to the Cry protein as the pest [17]. Accordingly, hereafter we differentiate this group as pest-related NTOs or prNTOs. It is important that regulators develop a reasonable understanding of the identity of prNTO insects, as well as the sensitivity and range of broader-sense NTOs that are likely to be exposed to the Cry toxin in the crop. In well-characterised and species-poor agro-ecosystems, this information may be available in the literature. In biodiverse environments, however, at least some *de novo* data-gathering will certainly be required.

When attempting to identify prNTOs and NTOs in a new area, it is useful to consider routes through which a beneficial prNTO could be exposed to the Cry toxin contained in a GM Cowpea plant. Crop herbivory provides direct exposure and this feature primarily differentiates the pests from the NTOs. Put simply, few organisms that feed extensively on a crop will also be afforded the status of a ‘beneficial’ NTO, especially if their presence significantly impacts on yield. However, exposure is also possible via bi- or multi-trophic exposure and through activities associated with pollination. Several studies have reported minimal or no adverse effects to predators or parasitoids of herbivores feeding on GM Bt crops [18], [19]. In contrast, pollinators can be directly exposed to the toxin if the Cry protein is present in the pollen and/or nectar [20]. Cry1ab is targets pests in the Lepidoptera [21]. Importantly, this group of insects is entirely herbivorous and so cannot be subject to bi-trophic exposure but given that many are pollinator nectarivores, some may be exposed to Cry1Ab during flower visitation. Thus, one key task is to identify prNTOs (i.e. from within this family) that are pollinator/nectar-thieves of cowpea flowers in Nigeria and so potentially exposed to the Cry1Ab endotoxin.

One source of difficulty resides in the high diversity of insect fauna in Nigeria [22] and the importance of surrounding vegetation in providing a locality-dependent source of pollinators for the crop. The potential for regional variability when coupled with the largely uncharacterised insect pollinator fauna of Nigeria means that manual identification of the prNTOs and other arthropod NTOs could be a protracted process that requires access to specialised entomologists and taxonomists. We seek to circumvent this problem by deploying DNA barcoding (species identification directly from DNA sequence at specified sites) as a more cost-effective means of species diagnosis using potential NTO pollinators/nectar thieves of GM Bt cowpea from sites throughout Nigeria as an exemplar for the approach.

## Results

We collected 163 insects from cowpea flowers across five agro-ecological zones (Table S1) and generated clean bidirectional COI barcodes for every individual. The resultant sequences ranged in size between 306 and 605 bp, and were trimmed to 306 bp to allow for alignment comparisons between all samples. The peaks of all trimmed electropherograms conformed to the Phred threshold (20) recommended for bi-directional DNA barcodes [23] (GenBank Accession Nos JQ733217–JQ733379).

**Figure 1:**
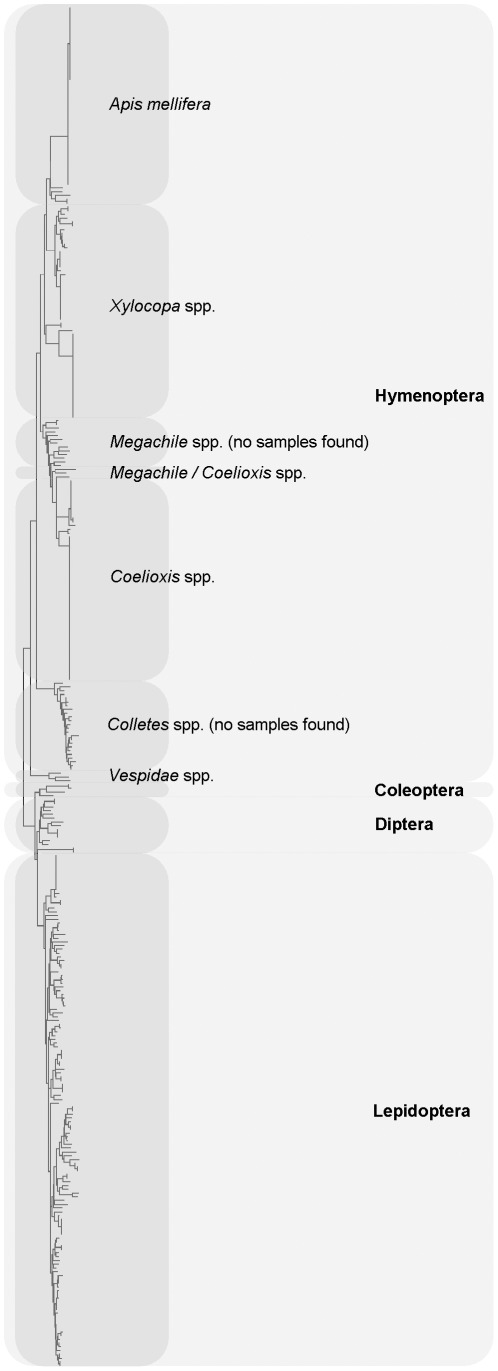
Insect diversity on cowpea flowers. Neighbour Joining tree representing COI barcodes for all species collected in the 7 different cowpea fields against barcode references downloaded from ncbi and CBOL. The tree has been build based on Kimura two-parameter distances (K2P) and 1000 bootstrap replications.

**Figure 2:**
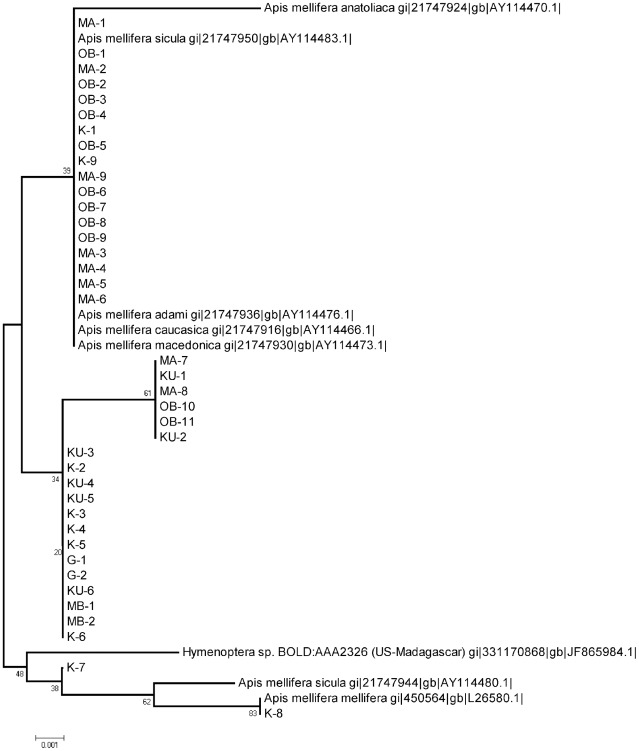
Honey bee clade collected from cowpea flowers in Nigeria. Neighbour Joining tree representing COI barcodes for *Apis mellifera* showing no clear separation between subspecies. The tree has been build based on Kimura two-parameter distances (K2P). The samples collected in Nigeria are specified using site code and samples number (e.g. MA-1 was collected MA: Mbano site A and 1 is the sample ID) see Table S1 for site code. Reference sequences have been downloaded from ncbi and CBOL and they indicate species name plus entry ID.

**Figure 3:**
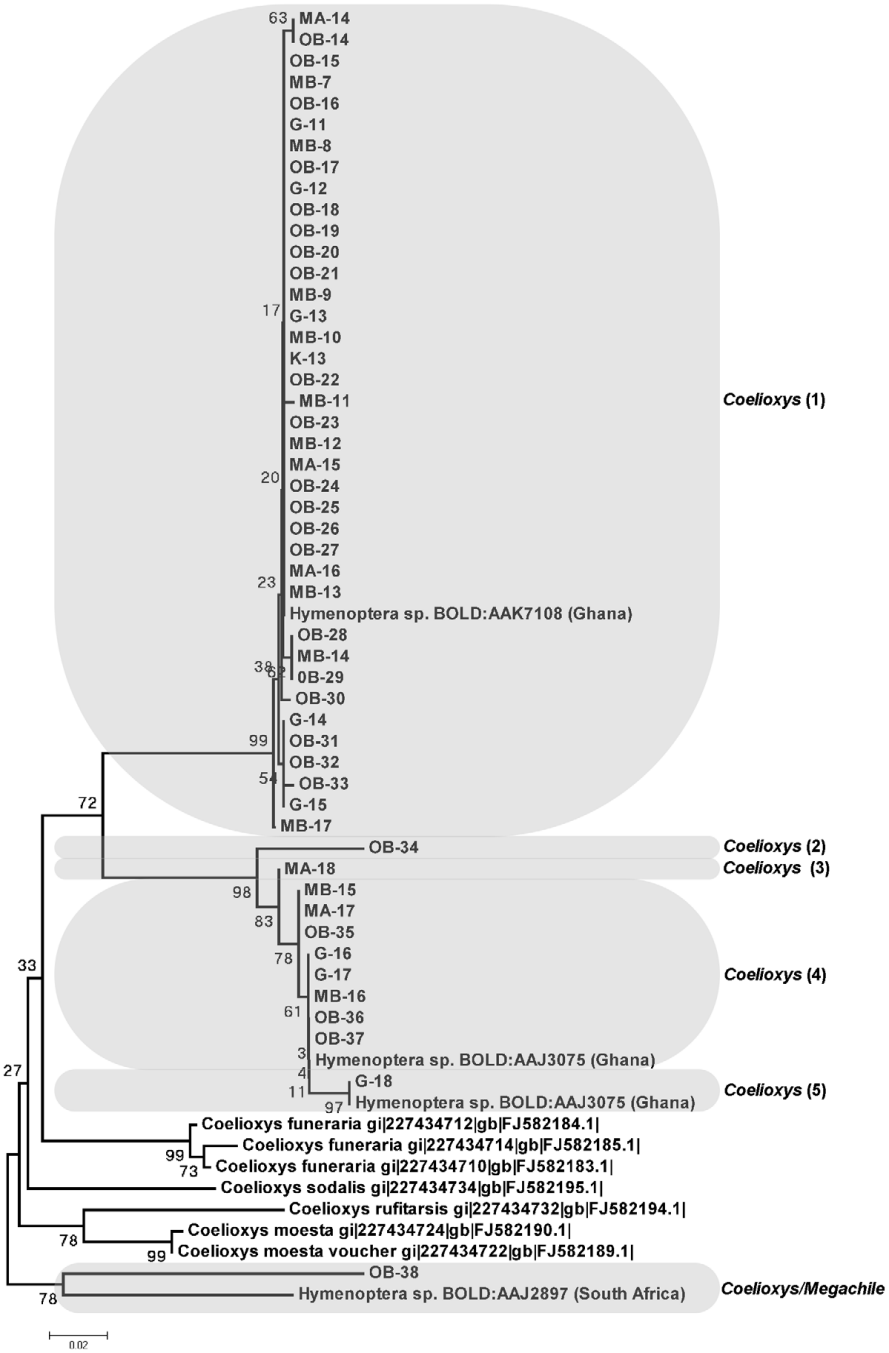
Cuckoo leaf-cutter bee clade collected from cowpea flowers in Nigeria. Neighbour Joining tree representing COI barcodes for individuals clustering in the *Coelioxys* clade. The tree is based on Kimura two-parameter distances (K2P). The five OTUs (presumed species) of *Coelioxys* collected in Nigeria are highlighted. Individuals specified using site code and sample number (e.g. MA-1 was collected MA: Mbano site A and 1 is the sample ID) see Table S1 for site code. Reference sequences have been downloaded from ncbi and CBOL and they indicate species name plus entry ID.

**Figure 4:**
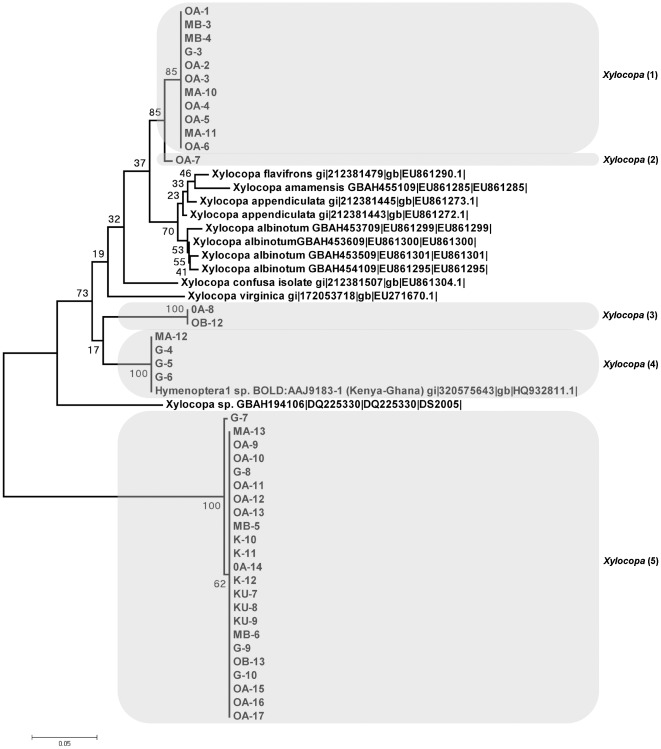
Carpenter bee (*Xylocopa*) clade collected from cowpea flowers in Nigeria. Neighbour Joining tree representing COI barcodes for *Xylocopa* clade showing separation into 5 distinct OTUs (presumed species). The tree is based on Kimura two-parameter distances (K2P). The five species of *Xylocopa* collected in Nigeria are highlighted and samples specified using site code and sample number (e.g. MA-1 was collected MA: Mbano site A and 1 is the sample ID) see Table S1 for site code. Reference sequences have been downloaded from ncbi and CBOL and they indicate species name plus entry ID.

**Figure 5:**
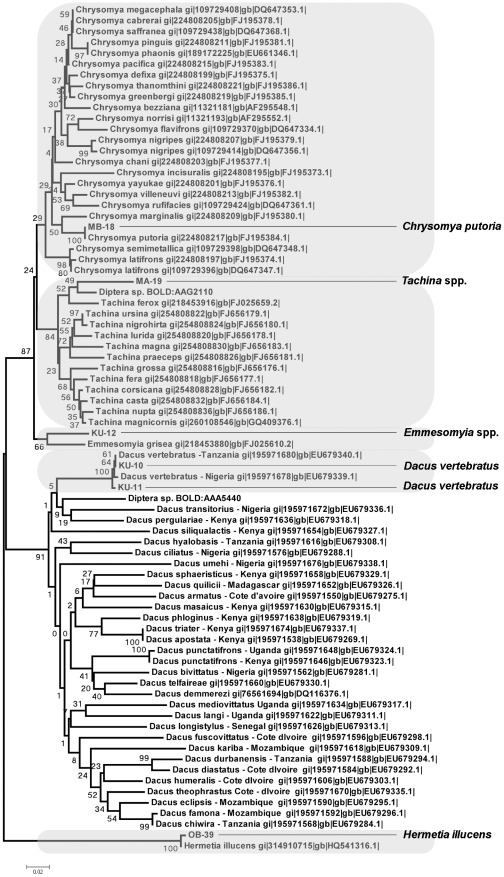
Insects in the Diptera clade collected from cowpea flowers in Nigeria. Neighbour Joining tree representing COI barcodes for Diptera showing species separation. The tree is based on Kimura two-parameter distances (K2P). The four species collected in Nigeria are highlighted and samples specified using site code and samples number (see Table S1). Reference sequences have been downloaded from ncbi and CBOL and they indicate species name plus entry ID.

**Figure 6:**
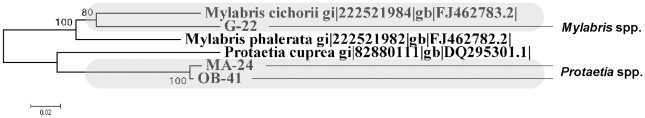
Insects in the Coleoptera clade collected from cowpea flowers in Nigeria. Neighbour Joining tree representing COI barcodes for Coleoptera showing species separation. The tree is based on Kimura two-parameter distances (K2P). The samples collected in Nigeria are highlighted and specified using site code and samples number (Table S1). Reference sequences have been downloaded from ncbi and CBOL and they indicate species name plus entry ID.

A single Neighbor Joining (NJ) tree featuring all field-captured insect COI sequences along with reference barcodes from ncbi and the CBOL databases comprised four major clusters corresponding to the Hymenoptera, Coleoptera, Diptera and Lepidoptera, and four subclusters within the Hymenopteran clade corresponding to bees within *Xylocopa*, *Apis*, *Coelioxys/Megachile* and *Vespidae* (Fig. 1). No anomalies were noted amongst any of these clades, with all ‘known’ reference samples clustering appropriately (Fig. 1). NJ trees were then generated for each cluster separately, and distances within and between species being calculated relative to barcode reference samples (Figs. 2, 3, 4, 5, 6). From these trees we were able to provisionally assign individuals to 31 nominal taxa, including 28 provisionally identified to genus, six to species and one to family (Table S2). Mean species divergence (K2P) was 0.648 and overall mean disparity index was 2.210; consistent with high species insect biodiversity and modest intraspecific variation.

The principal pollinators, bees, constituted 80% (130/163) of the individuals collected. These insects divided into 13 groups, with 12 resolving to species or genus rank (Table S2). As in previous studies of West African cowpea pollinators [24], [25], honey bees (*Apis melifera*; Fig. 2), cuckoo leaf-cutter bees (five clusters within *Coelioxys*; Fig. 3) and carpenter bees (five clusters within *Xylocopa*; Fig. 4) were all highly represented among the captured individuals. The Dipteran Clade comprised five subclades containing at least one field-collected individual (Fig. 5). Four specimens could be identified with some confidence to species level (two individuals of *Dacus vertebrates*, and one each of *Chrysomya putorya* and *Hermetia illucens*), with one of the remaining two individuals falling within a subclade comprising various *Tachina* reference sequences and so being assigned to that genus. The final individual was tentatively identified as belonging to *Emmesomyia* on the basis of its closest match. There were just two individuals collected in the Coleoptera clade (Fig. 6). One sample clustered between two reference sequences for *Mylabris* and so was assigned to this genus. The other two specimens generated very similar sequences that clustered most closely with a *Protaetia* species. They were therefore tentatively assigned to this genus.

**Figure 7:**
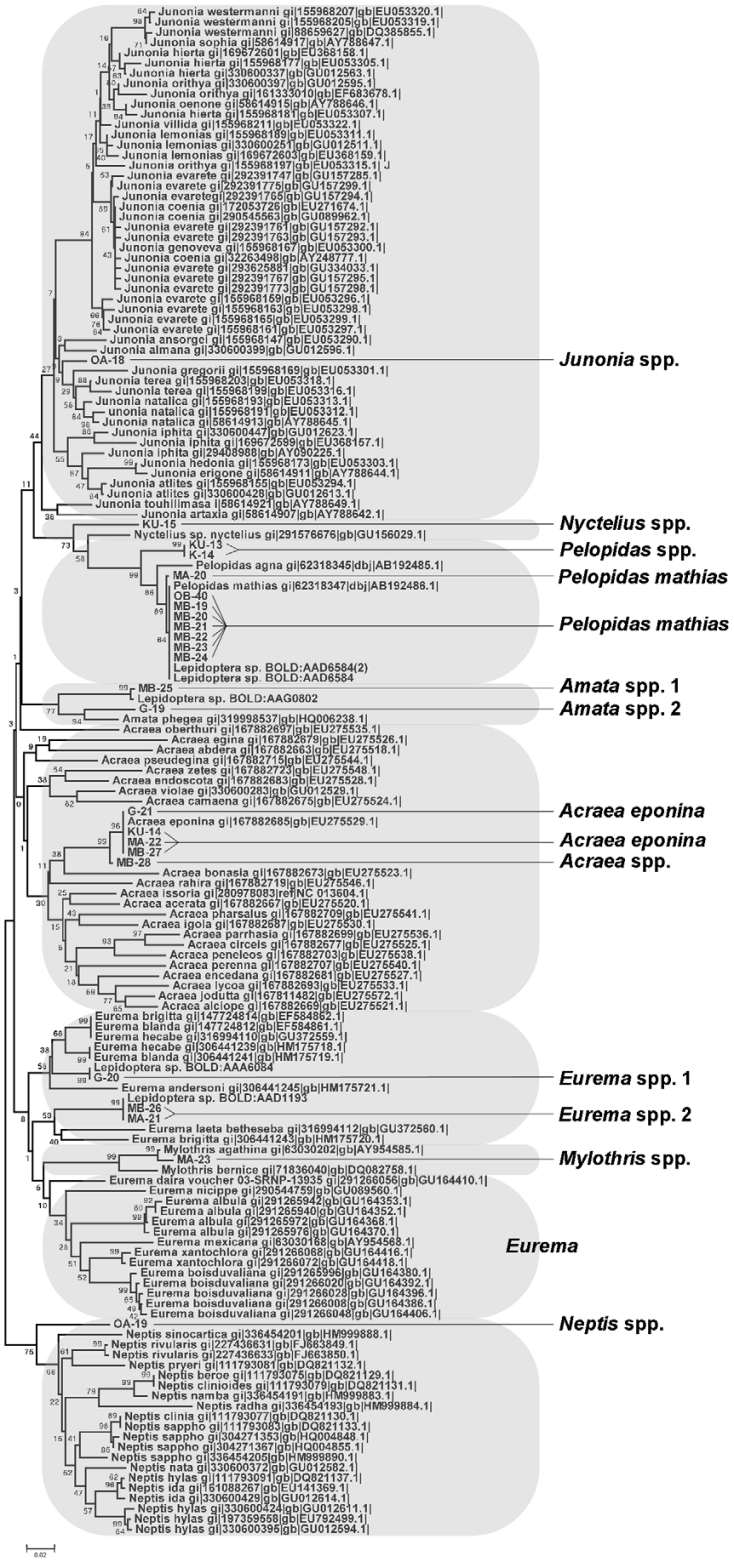
Insects in the Lepidoptera clade collected from cowpea flowers in Nigeria. Neighbour Joining tree representing COI barcodes for Lepidoptera showing clear separation according to genus and species. The tree is based on Kimura two-parameter distances (K2P). The samples collected in Nigeria are highlighted and specified using site code and samples number (see Table S1). Reference sequences have been downloaded from ncbi and CBOL.

When considering the prospective environmental risks posed by the release of a GM *Bt Cry1AB* cowpea, attention should focus primarily on the Lepidopteran NTOs since these are most likely to be both Cry1A-susceptible and also valued as pollinators of the crop and/or of other plant species. Reference to the Lepidopteran NJ tree revealed field-collected specimens clustered with reference samples from eight genera (*Eurima*, *Amata*, *Pelopidas*, *Neptis*, *Mylothris*, *Acraea*, *Nyctelius/Coeliades* and *Junonia*) (Fig. 7), a diagnosis that was concordant with the independent phenotypic diagnoses (Figure S1). There were eight exact sequence matches for *Pelopidas mathias* and 4 for *Acraea eponina*. The capacity to assign species binomials to some of the residual specimens was slightly impaired by the only partial release of information relating to the taxonomic identity of reference barcode sequences held on the BOLD database. For instance, there were two samples in a larger cluster of ncbi sequences identified as *Eurima* that were each identical to a different unnamed barcode reference sequence held on the BOLD database. In these cases, the samples were tentatively identified as *Eurima*. Likewise, another sample was identical to another BOLD reference sequence a clustered with similar ncbi sequences labelled as *Amata*. In these cases the BOLD barcode sequences currently precluded species identification, although this will change once the references are made fully available. Thus, in the majority of cases it was possible to at least provide provisional genus names on the basis of supported co-clustering with named ncbi database sequences. Assignment to genus was viewed as more tentative in two cases *Nyctelius* and *Neptis*, where the samples were relatively well separated from the nearest neighbour reference; a divergence supported by 100% bootstrap values. In the case of the former, morphological identification was provisionally assigned to *Coeliades* and so uniquely, did not conform to barcode analysis.

Members of the Coleoptera are unlikely to be targeted by Cry1Ab but are likely to be susceptible to Cry3 proteins. They were therefore also deemed to be of potential interest for subsequent submissions of GM cowpea events containing Cry3 proteins (targeting Coleopteran pests).

## Discussion

Continuing concern over African Food Security has led many authors to propose that cultivation of GM crops is a vital part of a broad suite of measures needed to address the problem [26]–[28]. However, the universal requirement for regulatory oversight of GM crop commercialisation has the potential to become a significant barrier to adoption in countries with less developed economies [29]. Viewed in this context, a vital challenge is to reduce the cost of environmental risk assessment without compromising efficacy or robustness of the process.

The global success of GM crops containing Cry proteins suggests that this trait will be among the first to be introduced into any region. One primary concern that applies to all receiving environments is the need to assess the potential for harm to NTOs, particularly to pollinators and other beneficial insects. There is a large body of evidence to indicate the activity of Cry toxins is largely restricted to their target taxonomic group (typically Order) [16], and tiered exposure experiments against a broad suite of sentinel species is generally regarded as sufficient to discount the possibility of harm outside species belonging to the targeted taxonomic group [30]. This line of reasoning has proved highly effective but cannot be used to discount possible harm to prNTOs. For the Cry proteins currently on the market, this issue primarily relates to members of the Lepidoptera (Cry1 and Cry2 proteins) and Coleoptera (Cry3 proteins) [15]–[17]. Both arthropod groups contain many useful pollinators that could be exposed to the toxin when foraging for pollen and/or nectar. Assembling a comprehensive list of potential pollinator NTOs from within these insect groups for any GM crop will help to identify prNTOs for the two types of Cry protein most likely to be introduced in the near future.

NTO identification is especially challenging in poorly characterised and biodiverse receiving environments, requiring appropriate entomologial expertise covering a wide taxonomic range coupled with the time-consuming process of morphological diagnosis. Incomplete coverage of taxonomic expertise typically leads to variability in the level of diagnosis reached between groups of insects. For example, in previous studies of African cowpea pollinators some individuals were identified to genus/specific rank whereas others were merely assigned to Order (e.g. Flies, Diptera [24], Dragonflies, Odonata [25] Beetles, Coleoptera [24]), Suborder (wasps, Apocrita [24]) or Family (Ants, Formicidae [24]). Thus, one of the two taxonomic groups of greatest interest (Coleoptera) was relatively poorly defined. This problem also applies for relatively well-worked invertebrate faunas where resources are more plentiful. For example, the UK farm scale evaluations [31] represent one of the most comprehensive and celebrated field studies of the impact of GM crops on the diversity of farmland invertebrates so far completed. However, even here the level of diagnosis achieved varied from species to order, depending on taxonomic group [31]. In the current study, we were able to use DNA barcoding to achieve better levels of sample diagnosis (typically to genus or species level) across all four Orders of arthropods captured on cowpea flowers across Nigeria. This level of diagnosis has value beyond providing a measure of overall or group-specific biodiversity, and is particularly useful from the perspective of problem formulation. For instance, whilst no arthopods are specifically listed as being of direct conservational importance to Nigeria [32], several of the genera identified do contain species that feature on the IUCN global red list [33]. Among the Lepidopteran genera identified from Nigeria in the present study, *Neptis* contains eight red-listed species, *Junonia* has five and *Mylothris* has four. Only four of the listed *Neptis* species are known to occur in Nigeria (*N. melicerta*, *N. nicoteles*, *N. occidentalis* and *N. quintilla*) and these all are described as being of ‘Least Concern’ [34]–[37]. Likewise, the three Junonia species recorded from Nigeria (*J. hierta*, *J. oenone*, *Precis rauana* = *J. rauana* ) are all listed as being of least concern [38]–[40]. The one listed *Mylothris* species from Nigeria (*M. asphodelus*) is also described as being of least concern [41]. Surprisingly perhaps, the one butterfly identified only to family rank (Skipper butterflies) also presented little problem since none of the thirteen species from seven genera in this family that were included on the IUCN list occur anywhere in sub-Saharan Africa [42]–[54]. Thus, despite patchiness in the taxonomic coverage of fully vouchered barcode sequences, it was nevertheless possible to derive roughly equivalent levels of identification across all groups. This was sufficient to deduce that on the basis of the known fauna, none of the captured specimens appeared to have designated conservational value for Nigeria nationally or were known to be at risk of extinction in a global sense. However, this overlooks the possibility that some of the specimens captured may represent hitherto undescribed or cryptic species. A more concerted programme would be required to address this possibility. Moreover, we maintain that a concerted effort to assemble vouchered reference barcodes for prNTOs for all crops in biodiverse regions would yield disproportionate benefits for the risk assessment process and could be assembled at comparatively trivial cost. A comprehensive list of potential invertebrate NTOs in a region would significantly cut the cost and enhance the power of ERAs by dramatically increasing the sample sizes that can be handled and reducing the time and physical resources needed for diagnosis. More importantly, a dataset of this kind would ensure equivalence of taxonomic usage, facilitate effective problem formulation for new events and allow direct comparisons between studies in the same or different areas. Furthermore, databases of this kind would hold generic value for multiple submissions. In the present study this point is best illustrated by the discovery of two Coleopteran genera on the flowers of cowpea (*Mylabris* and probably *Protaetia*; Figure 7) that would probably be susceptible to future cowpea events containing Bt (Cry3) proteins. Of these only the latter contains a single IUCN listing (*P.sardia*) which does not occur in sub-Saharan Africa [55].

Curiously, we did note that one of the Lepidopteran species (*Phelopidas mathias*) captured on cowpea flowers (8 individuals) is also a significant pest of rice [56]. Thus, it appears that possible negative effects of Cry1Ab on this NTO which is benign on cowpea may actually confer positive benefits to another crop (rice). Close examination of samples from the Dipteran tree (Figure 6) revealed another potential NTO *Dacus vertebratus* that is a known pest of fruit crops [57]. However, the Cry1Ab protein has been shown to have little effect on other Dipteran species [58] and so is unlikely to significantly affect this species.

Overall, we feel the use of COI barcoding for NTO discovery is rapid, cheap, technically undemanding and above all, will generate results that are reproducible between studies. Furthermore, despite development of non-destructive anatomical examination protocols for some taxa, morphological identification of arthopods frequently requires destructive removal of internal organs for reliable diagnosis [59]. In these cases, it may not be possible to verify identification at a later date through re-examination of the same specimen, should the accuracy of diagnosis be in doubt or else should taxonomic revision generate fresh ambiguity (e.g. discovery of a cryptic species or the inflation of a subspecies to specific rank). In contrast, DNA barcode information is well-suited to such post-hoc corrections. Certainly, several studies have reported that divergence in DNA barcode sequence subsequently coincided with the discovery of cryptic morphological species [60]. This possibility has particular value when attempting to characterise possible NTOs in a biodiverse and understudied fauna. Moreover, specimens that fail to cluster with known reference sequences can either be targeted for close taxonomic examination or else provisionally assigned a working taxonomic status (e.g. unknown member of a particular genus or tribe) which may be sufficient for certain regulatory decision-making purposes. For example, in the present work, there were two distinct field sequences with bootstrap support residing within clusters associated with reference Lepidopteran sequences representing the genera *Pelopipas*, *Amata*, *Acraea* and *Eurema* (Fig. 2). For risk scenarios relating to unwanted changes to pollinator function or overall biodiversity, in this case assignment to genus is probably sufficient for decision-making purposes. However, had any of the genera be deemed to be of exceptionally high conservational, ecological or scientific interest (e.g. had they have been endemics or represented isolated monophyletic taxa), then closer examination of these samples may have been deemed necessary, even though this issue may not have come to light had identification been based entirely on morphological grounds.

To conclude, we argue that the assembly of reference barcodes for invertebrate pests and NTOs would reduce unnecessary duplication of effort between studies, identify region-specific issues and reduce the time and material costs of compiling and evaluating submissions in biodiverse agri-ecosystems. Many have argued that sub-Saharan Africa has a pressing need to adopt GM technology if it is to address its ever-growing food security problem [61]. Given the prerequisite requirement for a cost-effective and robust environmental risk assessment (ERA) framework before adoption, this approach offers disproportionate benefits to the region by reducing time and financial resources required to compile ERAs in areas of high species richness.

## Materials and Methods

### Sampling and identification of samples

Samples of insects were collected from the interior of seven cowpea fields in seven locations across the five major agro-ecological regions in Nigeria (Table S1). The collections were made between the third week in June and second week in July, 2010. All necessary permits were obtained for the described field studies, (Federal Ministry of Environment, Nigeria. Kagoro Farm, Kontagora. Ogadu Farm, Umuodagu). The fields selected were largely restricted to the three varieties (Ife brown, SAMPEA7 and IT97K-499-35). Sampling spanned the entire cowpea-growing areas of the country. The areas covered by the study, their distances from Abuja and grid references were as follows: (i) Gombe (Northeast – Sahel savannah/arid/semi arid): 424 Km, 10°25′05″N, 11°19′50″E [PQS 22302 52360] (1 field) (ii) Kontagora (Northwest – Sudan savanna): 265.6 Km, 10°30′02″N, 05°32′57″E (one field) (iii) Kuje (Middle belt – Guinea savanna): 32.46 Km, 08°57′39″N, 07°40′45″E (1 field) (iv) Mbano/Umuduru (Southeast – Tropical rainforest): 917.17 Km, M_1_: 05°38′08″N, 07°14′55″E and M_2_: 05°40′08″N, 07°11′44″E (2 fields) and (v) Ogbomosho (Southwest – Guinea savanna): 370.12 Km, 0_1_: 08°14′02″N, 04°15′29″E and 0_2_: 08°10′27″N, 04°20′17″E (2 fields) (Table S1). Insects were collected from cowpea flowers either by direct capture into sterile containers or else using a sweep net and then decanting samples into plastic containers. All samples were killed immediately upon collection. Morphological identification of the Lepidopteran samples was performed by Dr Neil Gale of The magic of life Butterfly House, Aberystwyth, UK. Photographic images were collected of the identified specimens.

#### Preparation of specimens and DNA extraction

DNA was extracted from all sampled insects using the Wizard SV 96 Genomic DNA purification System (Promega, UK) according to manufacturers' instructions. Two insect forelegs were used for each DNA extraction. One additional starting step was added from the manufacture protocol to increase DNA yields:, an extra mechanical disruption of forelegs was performed prior to the chemical digestion. Insect material was then placed in a 2 ml 96 well rack with 0.5 mm tungsten beads and snap-frozen in liquid nitrogen before being mechanically ground using a TissueRuptor (Qiagen, UK) at high speed (20–30 Hz) for 2 min. Upon completion, 275 µl of Digestion solution Master Mix was added to each powder sample and incubated overnight (16 h) at 55°C as described in the manufactures' protocol.

DNA purification using the Wizard SV 96 Genomic DNA purification System was performed using a 96-well vacuum filtration system (Vac-Man 96 Vacuum Manifold). All reagents for DNA extraction were used at working concentration as provided in the kit, with exception of proteinase K (Promeg, UK), liquid nitrogen and 95% ethanol. The Wizard SV Wash Buffer was supplied as concentrate and needed dilution in 95% ethanol to achieve working concentration.

#### PCR conditions and sequences analysis

Polymerase chain reactions (PCR) were performed in MJ Research PCT100 (BioRad, UK), with individual sample wells comprising 20 µl reaction volumes that contained: 10 µl Taq-polymerase (BioMix), 2 µl of each of the forward and reverse primers, 4 µl of Nuclease-free water and 4 µl of mtDNA. For PCR amplification, the nucleotide sequences for the primers used were: *COX1* LEP(F1), 5′-ATTCAACCAATCATAAAGATATTGG-3′: LEP(R1), 5′-TAAACTTCTGGATGTCCAAAAAATCA-.3′. These universal primers are designed to amplify ∼600 base pairs of the CO1 region across a wide range of taxa (Hebert *et al.*, 2004).

The amplification protocol comprised: 5 cycles of 94°C/120 s, 94°C/40 s, 45°C/40 s, 72°C/60 s, 35 cycles of 94°C/40 s, 51°C/40 s, 72°C/60 s, and a final 5 min step at 72°C. The initial five cycles was set at a lower annealing temperature to strengthen amplification. All products were fractionated by agarose gel electrophoresis and post-stained with ethidium bromide. Strong amplicons of the appropriate size were then sent to the IBERS central sequencing facility (Aberystwyth University) for Sanger sequencing [62] using the CO1 barcoding sequencing protocol described by [60]. The resultant clean bi-directional sequences were trimmed to 477 bp (the maximum conserved length all samples) for alignment comparisons using Clustal.

#### Phylogenetic tree construction

The *cox1* sequences obtained from field-collected samples were compared with reference sequences secured from ncbi and CBOL databases to effect putative identifications for each insect sample. Phylogenetic trees were constructed from all barcode sequences by the neighbor joining method and parsimony using the Kimura 2-parameter model (NJ K2P) [63] (1000 bootstrap replicates) on the MEGA4 software [64]. NJ trees were then generated for each taxonomic cluster separately, and distances within and between species being calculated relative to barcode reference samples as described by Hebert et al [60]. Position of field-collected insects on the tree was then compared with those of co-clustering reference samples from the two databases.

## Supporting Information

Figure S1 **Images of Lepidoptera capyured on Cowpea flowers in Nigeria.** All individuals diagnosed on the basis of phenotype by Dr Neil Gale, The magic of life Butterfly House, Aberystwyth, UK. Images of some specimens used for COI barcoding are shown.
(DOCX)

Table S1 **Samples of insects were collected from the interior of seven cowpea fields in seven locations across the five major agro-ecological regions in Nigeria.** The collections were made between the third week in June and second week in July, 2010.
(DOCX)

Table S2 **Identity of insects visiting cowpea flowers in Nigeria.** Cowpea visitors collected in 7 different fields in Nigeria and identified using molecular barcoding sources (COI1) and morphological identification.
(DOC)
