# Supplementary figures and images for: DNA Barcoding Simplifies Environmental Risk Assessment of Genetically Modified Crops in Biodiverse Regions
Source: PLoS One. 2012 May 2;7(5):e35929. doi: 10.1371/journal.pone.0035929 (PMC3342289; doi:10.1371/journal.pone.0035929)

**Supporting information**

**Figure S1**.


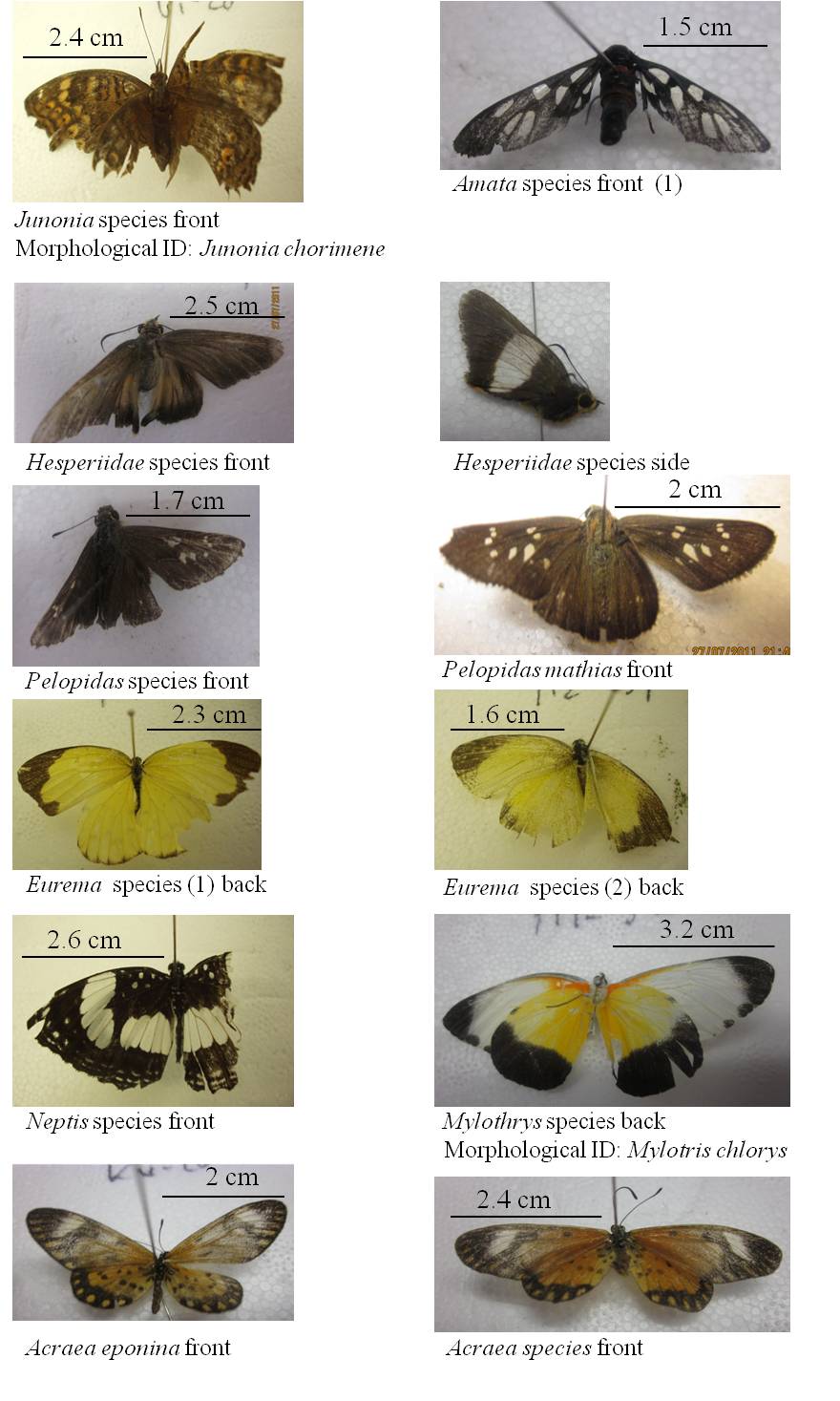

Supplement: Figure S1 — Images of Lepidoptera capyured on Cowpea flowers in Nigeria. All individuals diagnosed on the basis of phenotype by Dr Neil Gale, The magic of life Butterfly House, Aberystwyth, UK. Images of some specimens used for COI barcoding are shown. (DOCX) [file pone.0035929.s001.docx]
