# Supplementary material for: DNA Barcoding Simplifies Environmental Risk Assessment of Genetically Modified Crops in Biodiverse Regions
Source: PLoS One. 2012 May 2;7(5):e35929. doi: 10.1371/journal.pone.0035929 (PMC3342289; doi:10.1371/journal.pone.0035929)
Supplement: Table S1 — Samples of insects were collected from the interior of seven cowpea fields in seven locations across the five major agro-ecological regions in Nigeria. The collections were made between the third week in June and second week in July, 2010. (DOCX) [file pone.0035929.s002.docx]

**Supporting information**

**Table S1.**

| **Crop location** | **Location code** | **Agroecological zone** | **Coordinates** |
| --- | --- | --- | --- |
| Mbano | MA | Tropical rain forest | 05^o^ 38’ 08” N 07^o^ 14’ 55” E |
| Mbano | MB | Tropical rain forest | 05^o^ 40’ 08” N 07^o^ 11’ 44” E |
| Kuje | KU | Guinea savannah | 08^o^ 57’ 39” N 07^o^ 40’ 45” E |
| Gombe | G | Guinea savannah | 10^o^ 25’ 05” N 11^o^ 19’ 50” E |
| Kontagora | K | Guinea savannah | 10^o^ 30’ 02” N 05^o^ 32’ 57” E |
| Ogbomosho | OA | Dried savannah | 08^o^ 14’ 02” N 04^o^ 15’ 29” E |
| Ogbomosho | OB | Dried savannah | 08^o^ 10’ 27” N 04^o^ 20’ 17” E |
