# Supplementary material for: DNA Barcoding Simplifies Environmental Risk Assessment of Genetically Modified Crops in Biodiverse Regions
Source: PLoS One. 2012 May 2;7(5):e35929. doi: 10.1371/journal.pone.0035929 (PMC3342289; doi:10.1371/journal.pone.0035929)
Supplement: Table S2 — Identity of insects visiting cowpea flowers in Nigeria. Cowpea visitors collected in 7 different fields in Nigeria and identified using molecular barcoding sources (COI1) and morphological identification. (DOC) [file pone.0035929.s003.doc]

**Table S2: Identity of insects visiting cowpea flowers in Nigeria.**

| **Order** | **Family** | **Genus** | **Species** | **Number of individuals** |
| --- | --- | --- | --- | --- |
| Hymenoptera | Megachilidae | *Coelioxys (1)* |  | 38 |
|  |  | *Coelioxys (2)* |  | 1 |
|  |  | *Coelioxys (3)* |  | 1 |
|  |  | *Coelioxys (4)* |  | 8 |
|  |  | *Coelioxys (5)* |  | 1 |
|  |  | *Coelioxys / Megachile* |  | 1 |
|  | Apidae | *Apis* | *mellifera* | 38 |
|  |  | *Xylocopa (1)* |  | 11 |
|  |  | *Xylocopa (2)* |  | 1 |
|  |  | *Xylocopa (3)* |  | 2 |
|  |  | *Xylocopa (4)* |  | 4 |
|  |  | *Xylocopa (5)* |  | 23 |
|  | Vespidae |  |  | 1 |
|  |  |  |  |  |
| Coleoptera | Meloidae | *Mylabris* |  | 1 |
|  |  | *Protaetia* |  | 2 |
|  |  |  |  |  |
| Diptera | Calliphoridae | *Chrysomya* | *putoria* | 1 |
|  | Tachinidae | *Tachina* |  | 1 |
|  | Anthomyiidae | *Emmesomyia* |  | 1 |
|  | Tephritidae | *Dacus* | *vertebratus* | 2 |
|  | Stratiomyidae | *Hermetia* | *illucens* | 1 |
|  |  |  |  |  |
| Lepidoptera | Nymphalidae | *Junonia* |  | 1 |
|  |  | *Neptis* |  | 1 |
|  |  | *Acraea* | *eponina* | 4 |
|  |  | *Acraea* |  | 1 |
|  | Arctiidae | *Amata* (1) |  | 1 |
|  |  | *Amata* (2) |  | 1 |
|  | Hesperiidae | *Pelopidas* | *mathias* | 8 |
|  |  | *Pelopidas* |  | 2 |
|  |  | *Nyctelius* (BLAST match) *Coeliades* (morphological ID) |  | 1 |
|  | Pieridae | *Eurema (1)* |  | 1 |
|  |  | *Eurema (2)* |  | 2 |
|  |  | *Mylothris* |  | 1 |
